# Identification of a novel polyomavirus from a marsupial host

**DOI:** 10.1093/ve/veac096

**Published:** 2022-10-06

**Authors:** Magdalena Dunowska, Matthew Perrott, Patrick Biggs

**Affiliations:** School of Veterinary Science, Massey University, Palmerston North 4410, New Zealand; School of Veterinary Science, Massey University, Palmerston North 4410, New Zealand; School of Veterinary Science, Massey University, Palmerston North 4410, New Zealand; School of Natural Sciences, Massey University, Palmerston North 4410, New Zealand

**Keywords:** possum, polyomavirus, *Betapolyomavirus*, WUKI clade, marsupial virus, virus taxonomy

## Abstract

We report the identification and analysis of a full sequence of a novel polyomavirus from a brushtail possum (*Trichosurus vulpecula*) termed possum polyomavirus (PPyV). The sequence was obtained from the next-generation sequencing assembly during an investigation into the aetiological agent for a neurological disease of possums termed wobbly possum disease (WPD), but the virus was not aetiologically involved in WPD. The PPyV genome was 5,224 nt long with the organisation typical for polyomaviruses, including early (large and small T antigens) and late (Viral Protein 1 (VP1), VP2, and VP3) coding regions separated by the non-coding control region of 465 nt. PPyV clustered with betapolyomaviruses in the WUKI clade but showed less than 60 per cent identity to any of the members of this clade. We propose that PPyV is classified within a new species in the genus *Betapolyomavirus*. These data add to our limited knowledge of marsupial viruses and their evolution.

## 1. Introduction

Polyomaviruses are small, non-enveloped viruses classified within the *Polyomaviridae* family (Moens et al. 2017b). They contain a DNA genome of about 5,000 base pairs (bp) in length. Two transcriptional regions (early and late) encode five to nine proteins. The early and late genes are transcribed in opposite directions and separated by a non-coding control region (NCCR), which includes an origin of DNA replication and other regulatory elements for promoters of both early and late genes (Moens et al. 2017b). Early genes encode large (L) and small (S) non-structural tumour antigen (TAg) proteins, which are involved in the dysregulation of cell cycle and, occasionally, cell transformation. Late genes encode three to four structural viral proteins (VP), with VP1 being the main component of the capsid (Moens et al. 2017a, 2017b). Some polyomaviruses encode additional early and late proteins including agnoprotein, alternative large T antigen open reading frame (ALTO), or open reading frame (ORF) X (Buck et al. 2016). Polyomaviruses are currently classified into eight genera (*Alphapolyomavirus*, *Betapolyomavirus*, *Deltapolyomavirus*, *Gammapolyomavirus*, *Epsilonpolyomavirus*, *Etapolyomavirus*, *Thetapolyomavirus*, and *Zetapolyomavirus*), with the last four created within the past 2 years to accommodate a growing number of novel polyomavirus sequences (https://talk.ictvonline.org/taxonomy/, accessed 22 March 2022). The family contains more than eighty species, which include thirteen viruses that infect humans (Moens et al. 2017b).

Most human polyomavirus infections are asymptomatic in immunocompetent hosts. Some, however, have been associated with serious disease or cancer, predominantly in immunocompromised individuals (reviewed in Moens et al. 2017a). Examples include involvement of JC polyomavirus (human polyomavirus 2) in progressive multifocal leukoencephalopathy, BK polyomavirus (human polyomavirus 1) in nephropathy following kidney transplantation, or Merkel cell polyomavirus in Merkel cell carcinomas [3]. As only sequence data are available for most other mammalian polyomaviruses, it is currently unknown whether or not these infections are associated with specific diseases. A recently discovered racoon polyomavirus has been linked to the development of brain tumours in free-ranging raccoons (Pesavento et al. 2016). The tumorigenic potential of polyomaviruses caused considerable concerns when it was discovered that Vero cells used for the production of early poliovirus vaccines were contaminated with simian virus 40 (SV40). Although this virus causes development of tumours when inoculated into hamsters, iatrogenic introduction of SV40 into the human population did not lead to an epidemic of cancer, indicating that SV40 infection on its own is probably not sufficient to induce tumorigenesis in people (Qi et al. 2011). Avian polyomaviruses, in contrast, are important causes of disease and death, particularly in young birds (Muller and Nitschke 1986; Johne and Muller 1998).

Polyomaviruses tend to be relatively host-specific. Analysis of human and animal polyomavirus sequences indicated that these viruses most likely co-evolved with their hosts over at least half a billion years through co-divergence and occasional recombination events (Buck et al. 2016). The advancement of molecular tools in the past 10–20 years has led to the detection of novel polyomavirus sequences in a variety of hosts including mammals, arthropods, fish, and birds (Buck et al. 2016). However, only two polyomaviruses have been described so far in the marsupial host (Tasmanian devil polyomaviruses 1 and 2), with a full sequence available only for the former (Chong et al. 2019). Both were detected in the faecal samples from Tasmanian devils by NGS. In addition, two polyomavirus-like viruses were detected in skin lesions of animals suffering from progressive mucocutaneous papillomatosis and carcinomatosis syndrome. They were somewhat unusual, as they encoded avian-like polyomavirus T antigens and papillomavirus-like structural proteins and are hence not classified as polyomaviruses (Woolford et al. 2007; Bennett et al. 2008).

The Australian brushtail possum (*Trichosurus vulpecula*) is a marsupial native to Australia. It was introduced into New Zealand in the 19th century and has since become a significant pest to the country’s ecosystem (Cowan and Tyndale-Biscoe 1997). A fatal neurological disease of possums termed wobbly possum disease (WPD) was first observed at the captive possum colony in Invermay in the South Island of New Zealand. Further investigation was driven by a desire to identify an agent that may be utilised for biological control of possums in New Zealand (Mackintosh et al. 1995). Early clinical signs of WPD in experimentally infected animals include inappetance, temperament changes, and altered responsiveness to environmental stimuli, followed by cachexia and the development of more apparent neurological deficits, including fine head tremors, ataxia, and occasionally presumed blindness (Perrott, Wilks, and Meers 2000c; Giles et al. 2016). Most experimentally infected animals died or were euthanised due to the severity of clinical signs. The disease was also identified in free-living possums in New Zealand (Perrott et al. 2000a). Despite research efforts in the mid to late 1990s, the causative agent remained unknown until sequences of a novel arterivirus were detected by next-generation sequencing (NGS) from tissues of affected possums about 15 years later (Dunowska et al. 2012). That virus was subsequently experimentally proven to be responsible for WPD (Giles et al. 2016). Serological evidence suggest that approximately 30 per cent of possums in both New Zealand and Australia have antibodies to a WPD-like virus, suggesting that not all natural infections are fatal.

Here, we describe the detection and sequence analysis of a novel marsupial polyomavirus obtained from New Zealand possums during the investigation of the aetiology of WPD (Dunowska et al. 2012).

## 2. Materials and methods

### 2.1 Next-generation sequencing

NGS and data analysis were performed in 2010 as described previously (Dunowska et al. 2012). Briefly, the standard inoculum (SI) that was prepared during the 1996 transmission studies and stored at −80°C was used as a starting material. The SI comprised a 10 per cent suspension of tissues (spleen, liver, and brain) from seven possums affected by WPD (Perrott, Wilks, and Meers 2000c). The SI was treated with nucleases in order to enrich it for viral nucleic acids before extraction of nucleic acids and cDNA synthesis. The cDNA/DNA was further amplified in a multiple-displacement amplification (MDA) reaction using the Illustra GenomiPhi V2 DNA amplification kit (GE Healthcare), phenol-chloroform extracted, ethanol precipitated, and submitted to the Massey Genome Service (Massey University, Palmerston North, New Zealand) for sequencing on an Illumina GAII_X_ Genome Analyzer. Following pipeline processing, the Illumina data were depleted of host sequences by mapping to a repeat masked version of the *Monodelphis domestica* genome (the closest available to that of *Trichosurus vulpecula)* using the BWA (Li and Durbin 2009), Bowtie (Langmead et al. 2009), and SSAHA2 (Ning, Cox, and Mullikin 2001) aligners. *De novo* contigs assembled with ABySS (Simpson et al. 2009) and Velvet (Zerbino and Birney 2008) were compared to viral sequences available in GenBank using BLAST algorithms.

### 2.2. Possum polyomavirus PCR

Specific primers (Table 1) targeting a 213-bp region within VP1 and a 399-bp region within LTAg were designed to confirm the possum polyomavirus (PPyV) sequence obtained from the NGS assembly. Each 10 µL PCR reaction consisted of 0.5 µM of each primer and 1 µL of template DNA in a Hot Start PCR master mix (Roche). Nucleic acids extracted directly from the SI or obtained as described above for NGS were used as a template for PCR reactions. The cycling consisted of a 10-min denaturation/enzyme activation step at 95°C, followed by forty cycles of denaturing (95°C for 15 s), annealing (60°C for 15 s), and elongation (72°C for 1 min), followed by a final extension at 72°C for 7 min. The products were visualised following electrophoresis through a 1-per cent ethidium bromide-stained agarose gel. The PCR products of the expected size were cut from the gel. The DNA was extracted from the gel slices and submitted for sequencing to the Massey Genome Service.

**Table 1.** Primer sequences.

Primer	Sequence (5ʹ-3ʹ)	Position (nt)	Expected product size (bp)
PPyV.Tag.F	GTACCAGGCCACCCCAGCCA	3396–3415	
PPyV.Tag.R	GAGGGGGACCCCACCACTCC	3794–3795	399
PPyV.VP1.F	CCCCAAGGCCTGGGCCTCAT	1722–1741	
PPyV.VP1.R	GTGGTCCCTCCACACCCCCA	1934–1915	213
PPyV.VP1.R1	TGTCCTGGGAACAACCAGCACCT	1879–1857	158

In addition, a small number of selected archival tissues (spleen or liver) from WPD-affected (*n* = 7) and clinically normal (*n* = 9) possums were tested for the presence of PPyV DNA in a nested PCR. The WPD-affected possums were used for 1996 transmission trials. The clinically normal possums comprised controls for the 1996 (*n* = 2) and 2013 transmission trials (*n* = 7) (Giles et al. 2016). Each 10 µL PCR reaction consisted of 0.2 µM of each primer and 1 µL of template DNA in a Hot FirePol PCR master mix (Solis Biodyne). The cycling consisted of a 15-min denaturation/enzyme activation step at 95°C, followed by thirty-five cycles of denaturing (95°C for 10 s), annealing (60°C for 10 s), and elongation (72°C for 20 s), followed by a final extension at 72°C for 5 min. Primers PPyV.VP1.F and PPyV.VP1.R were used in the primary reaction, and PPyV.VP1.F and PPyV.VP1.R1 were used in the nested reaction (Table 1).

### 2.3. PPyV sequence analysis

The polyomavirus sequence was analysed and annotated using Geneious software version 9.1.8 (https://www.geneious.com). The positions of genes were predicted using the Geneious ORF finder. The splice sites for the expression of LTAg were predicted using the Splice Site Prediction by Neural Network tool (http://www.fruitfly.org/seq_tools/splice.html) and compared with other polyomavirus genomes. Phylogenetic analyses were performed using the nucleotide sequence of LTAg. Representative sequences of polyomaviruses were retrieved from GenBank based on accession numbers specified in the *Polyomaviridae* taxonomy profile published by the International Committee for Taxonomy of Viruses (Moens et al. 2017b). The sequences (*n* = 113) were aligned using the MUSCLE aligner within Geneious software, and phylogenetic trees were inferred using the maximum likelihood method in MEGA 11 software (Tamura, Stecher, and Kumar 2021). Pairwise alignments of nucleotide sequences of LTAg of selected polyomaviruses from *Betapolyomavirus* genus were performed using the SDT Virus Classification Tool (Muhire, Varsani, and Martin 2014).

## 3. Results

### 3.1. NGS assembly and confirmatory PCR

**Figure 1.**
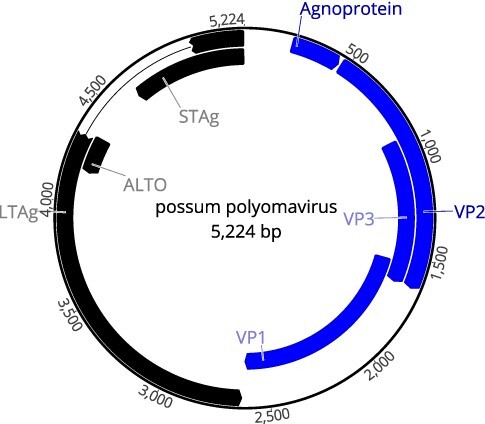
Genome organisation of PPyV. The predicted positions of early genes coding for large (LTAg) and small (STAg) T antigens, genes coding for viral structural protein (VP) 1, 2, and 3, and putative accessory genes (agnoprotein and ALTO) are indicated in black (reverse frames) or blue (forward frames).

A full genomic sequence of PPyV was obtained from the NGS assembly. The virus genome was 5,224 nt long with the organisation typical for polyomaviruses (Fig. 1). The two PCR reactions targeting VP1 and LTAg produced products of expected sizes with 100 per cent identity to the NGS assembly. Confirmatory PCR reactions produced expected products only when MDA-amplified nucleic acids that had been enriched for viral sequences were used as a template. No bands were visible in PCR reactions with non-enriched nucleic acids from the SI, either with VP1 primers or with LTAg primers.

### 3.2. Nested PPyV PCR

PPyV DNA was amplified from only 1/16 archival samples from a clinically normal (non-WPD) possum.

### 3.3. PPyV genome analyses

The predicted organisation of the genome of the novel PPyV was typical for polyomaviruses, including early (LTAg and STAg) and late (VP1, VP2, and VP3) coding regions separated by NCCR of 465 nt (Fig. 1 and Table 2). The NCCR contained the predicted origin of replication and regulatory sequences for early and late transcriptions (DeCaprio and Garcea 2013). The predicted early proteins (LTAg and STAg) contained several conserved motifs described for other polyomaviruses (Table 3). In addition to a putative agnoprotein gene (nt 222–458), another ORF was predicted in this region that may be expressed from a non-AUG initiation codon (nt 262–453) (Cao and Slavoff 2020). Finally, an alternative T ORF (ALTO)-like ORF at nt positions 4154–4342 and a hypothetical ORF of an unknown function at nt positions 4607–4326 were identified.

**Table 2.** Predicted coding regions of PPyV.

Gene	Start	Finish	Length (bp)	Direction	Size of protein product (aa)	Size of protein product (kDa)	Isoelectric point
Agnoprotein	222	458	237	Forward	78	8.7	11.40
Viral Protein 2	466	1,644	1,179	Forward	392	41.8	4.83
Viral Protein 3	913	1,644	732	Forward	243	27.1	9.76
Viral Protein 1	1,535	2,617	1,083	Forward	360	39.7	7.95
ALTO	4,342	4,154	192	Reverse	63	7.4	12.65
Large T antigen	5,224^a^	4,973	1,950	Reverse	649	75.1	6.68
	4,325	2,628					
Small T antigen	5,224	4,634	591	Reverse	196	23.5	9.64

a Predicted to be expressed over two intervals.

**Table 3.** Conserved motifs identified in predicted proteins of PPyV.

Protein	Name	Motif	Amino acid position	References
Large T antigen	PP2A-B′ binding site	[LM]xx[ILV]xE	13–18, 55–60	Wu et al. 2017
	DnaJ	HPDKGG	42–47	Pipas 1992
	YG(S/T)	YGS/T	88–90	Houben et al. 2015
	pRB1	LXCXE	107–111	Pipas 1992
	TPPK	TPPK	125–128	DeCaprio and Garcea 2013
	NLS^a^		126–137	Pipas 1992
	T antigen Ori binding		136–253	
	DNA-binding domain A	SNRT	151–154	Johne et al. 2006
	DNA-binding domain B	HRVSA	202–206	Johne et al. 2006
	Zinc finger motif	CX_2_CX_7_HX_3_H	304–319	
	ATPase	GPX3GKT	426–433	
	ATPase	GX3VNLE	502–509	
	CR1^b^	LXXLL	574–578, 575–579	Pipas 1992
Small T antigen	PP2A-B′ binding site	[LM]X_2_[ILV]XE	13–18, 55–60	Wu et al. 2017
	DnaJ	HPDKGG	42–47	Pipas 1992
	Zinc-binding domain	C*X*_7,8_C*X*C*X*_2_C*X*_5_H*X*_15,16_C*X*C*X*_2_C	125–165	Cho et al. 2007; Mateer, Fedorov, and Mumby 1998

a The putative nuclear localisation signal (NLS) was predicted using the NLS-prediction algorithm, available at http://nls-mapper.iab.keio.ac.jp/cgi-bin/NLS_Mapper_form.cgi.

b This CR1 motif is conserved in the N-terminal part of the Large TAg in other polyomaviruses.

### 3.4. Phylogeny

**Figure 2.**
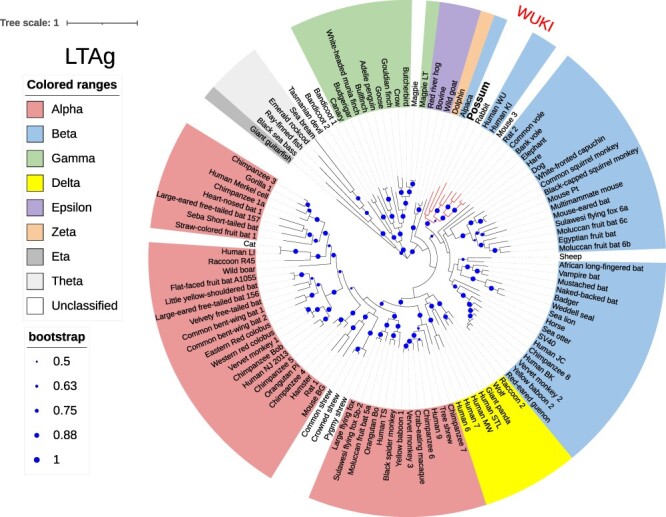
The evolutionary history was inferred by using the maximum likelihood method and Le_Gascuel_2008 model (Le and Gascuel 2008) using 113 amino acid sequences of large T antigen (LTAg). The tree with the highest log likelihood (−108,037.47) is shown. Bootstrap support (shown as proportions on a scale 0–1) higher than 50 per cent is shown as dark blue circles that are proportional to the percentage of trees in which the associated taxa clustered together. Initial tree(s) for the heuristic search were obtained automatically by applying Neighbor-Join and BioNJ algorithms to a matrix of pairwise distances estimated using the Jones-Taylor-Thornton (JTT) model and then selecting the topology with the superior log likelihood value. The tree is drawn to scale, with branch lengths measured in the number of substitutions per site. There were a total of 1,395 positions in the final dataset. Evolutionary analyses were conducted in MEGA11 (Tamura, Stecher, and Kumar 2021), and the tree was drawn using an Interactive Tree of Life (iTOL) programme (Letunic and Bork 2021). The branches are labelled with virus nicknames (common name of the host plus an additional identifier for multiple viruses from the same host). Full virus names and the accession numbers are listed in the Supplementary file S1. The taxonomic classification into one of the eight genera of Polyomaviridae family is indicated by the colour corresponding to the Greek alphabet letter that forms a prefix for that genus. PPyV clustered among betapolyomaviruses within the WUKI clade (labelled, with branches shown in red).

**Figure 3.**
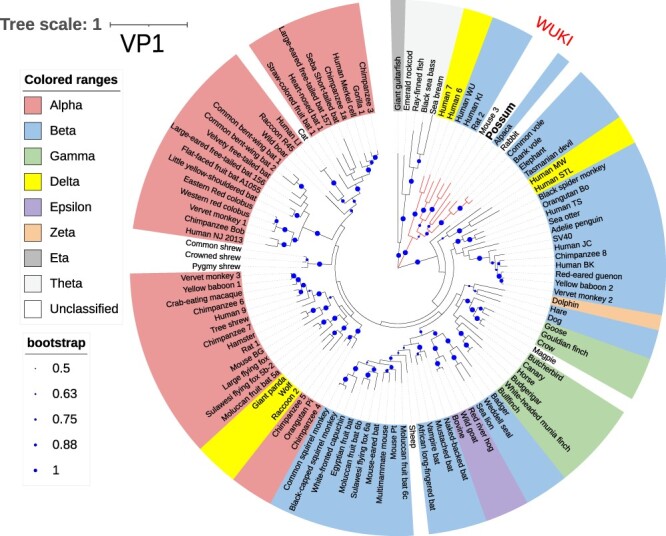
The evolutionary history was inferred by using the maximum likelihood method and Le_Gascuel_2008 model (Le and Gascuel 2008) using 111 amino acid sequences of Viral Protein 1 (VP1). The tree with the highest log likelihood (−53,187.61) is shown. Bootstrap support (shown as proportions on a scale 0–1) higher than 50 per cent is shown as dark blue circles that are proportional to the percentage of trees in which the associated taxa clustered together. Initial tree(s) for the heuristic search were obtained automatically by applying Neighbor-Join and BioNJ algorithms to a matrix of pairwise distances estimated using the JTT model and then selecting the topology with the superior log likelihood value. The tree is drawn to scale, with branch lengths measured in the number of substitutions per site. There were a total of 663 positions in the final dataset. Evolutionary analyses were conducted in MEGA11 (Tamura, Stecher, and Kumar 2021) and the tree was drawn using an Interactive Tree of Life (iTOL) programme (Letunic and Bork 2021). The branches are labelled with virus nicknames (common name of the host plus an additional identifier for multiple viruses from the same host). Full virus names and the accession numbers are listed in the Supplementary file 1. The taxonomic classification into one of the eight genera of Polyomaviridae family is indicated by the colour corresponding to the Greek alphabet letter that forms a prefix for that genus. PPyV clustered among betapolyomaviruses within the WUKI clade (labelled, with branches shown in red).

**Figure 4.**
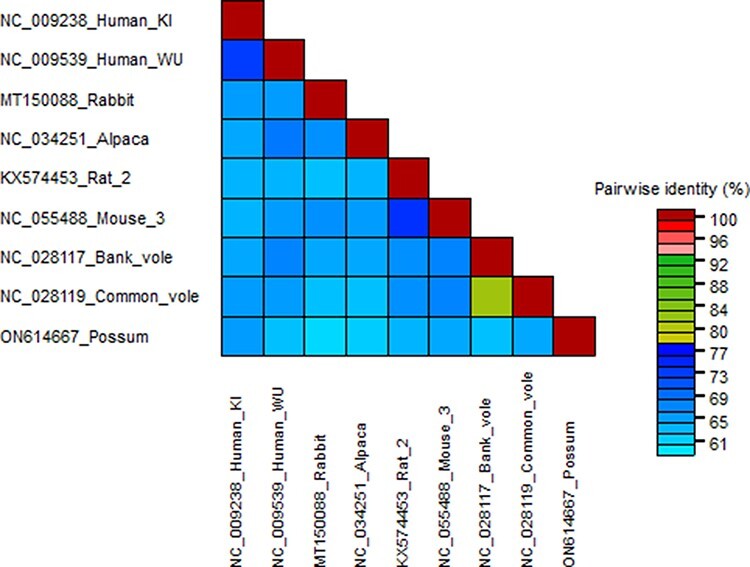
Percent identity between large T antigen (LTAg) nucleotide sequences of polyomaviruses from the WUKI clade that clustered together with PPyV in the maximum likelihood phylogenetic tree (Fig. 2). The accession numbers are listed next to the virus nicknames used in Figs 2 and 3. The figure was generated using SDT Virus Classification Tool (Muhire, Varsani, and Martin 2014).

The PPyV was clustered within the WUKI group of betapolyomaviruses both in the LTAg (Fig. 2) and VP1 tree (Fig. 3). The pairwise identity scores between LTAg gene sequences of PPyV and eight polyomaviruses from the WUKI cluster ranged from 59 to 64 per cent (Fig. 4).

## 4. Discussion

Therian mammals are divided into two subclasses: eutherian (placental) mammals and marsupials. In contrast to a large number of viruses that infect eutherian mammals, very few marsupial viruses have been identified thus far (Harding et al. 2021). The only viruses identified from the possum host include WPD virus (Dunowska et al. 2012; Giles et al. 2016), two enteroviruses (Zheng 2007), an adenovirus (Thomson, Meers, and Harrach 2002), a papillomavirus (Perrott et al. 2000b), and an endogenous Type D retrovirus (Baillie and Wilkins 2001). In addition, herpesvirus- and coronavirus-like particles have been observed in possum faeces using electron microscopy but not characterised further (Rice and Wilks 1996). As such, the identification of a novel polyomavirus from naturally infected possums expands the range of known possum viruses and provides an important contribution to our limited knowledge of marsupial viruses. The sequence of the novel polyomavirus was obtained from tissues of possums affected by WPD. However, polyomavirus sequences were not amplified by PCR from nucleic acids extracted from the SI without prior enrichment for viral sequences and MDA amplification, suggesting that the levels of PPyV RNA in the SI were low. As the SI contained at least 10^5^ possum-infective doses of the WPD agent and was highly infectious (Perrott, Wilks, and Meers 2000c), the low levels of PPyV nucleic acids in the SI were incompatible with its aetiological involvement in WPD. This is consistent with the fact that mammalian polyomaviruses have not been typically linked to severe disease in immunocompetent hosts, although a possible link between bovine polyomavirus 2 infection and non-suppurative encephalitis in cattle has been recently suggested (Hierweger, Koch, and Seuberlich 2020). The fact that PPyV was not amplified from any of the seven archival samples from WPD-affected possums (all of which contained nidovirus RNA) further supports the coincidental presence of PPyV in the SI.

It remains to be determined whether PPyV infection is associated with any disease in the possum host. By comparison, there is currently no clear association between infection with human polyomaviruses that clustered with PPyV (WU or KI) and disease. Despite the high prevalence of infection with either virus worldwide (Nguyen, Le, and Wang 2009), they have been only occasionally detected in the respiratory tract of individuals with acute respiratory disease (Babakir-Mina et al. 2013). The information about other mammalian viruses from the WUKI clade is sparse and mostly limited to the description of novel viral sequences.

Livers from six possums that were inoculated with the SI during prior transmission studies were negative for PPyV DNA when tested by nested PCR, indicating that these possums were either not infected with PPyV as a result of SI inoculation or that livers were not the optimal sample type to be tested. The low levels of PPyV in the SI are consistent with the former. We cannot fully exclude an alternative possibility that the SI got cross-contaminated at some point with a virus that originated from a different species, but we consider this unlikely for several reasons. First, most likely, not all of the seven possums that contributed tissues to the SI were infected with PPyV, which would have contributed to the ‘dilution effect’ of the mixed sample. This may explain low levels of PPyV DNA in the SI. In addition, the PPyV-positive tissues included in the SI may have been persistently, as opposed to actively, infected with PPyV. One would expect lower levels of viral DNA in persistent compared with active infection, although our current understanding of polyomavirus persistence is limited (Imperiale and Jiang 2016). Second, the tissues that were used for the preparation of the SI (liver, spleen, and brain) are expected to be sterile, and hence, any pathogens detected are more likely to represent true infection rather than environmental contamination. The samples were collected aseptically, which further minimised the possibility of contamination to occur post mortem. Third, PPyV DNA was detected by nested PCR in a spleen sample of a possum that was caught in the wild about 17 years after the SI was prepared. As none of the other fifteen possums tested by PCR were positive for PPyV DNA, this is unlikely to represent cross-contamination during the PCR set-up.

Polyomaviruses have been classified into eight genera based on the amino acid sequence of LTAg (Moens et al. 2017b). The hosts for alpha-, beta-, delta-, zeta-, and epsilonpolyomaviruses are mammals, gammapolyomaviruses infect birds while eta- and thetapolyomaviruses have been detected so far only from fish. Both the LTAg and VP1 phylogenetic trees showed expected topologies, with members of the same genera clustering together in the LTAg tree (Buck et al. 2016; Moens et al. 2017b). The position of PPyV within the WUKI clade of betapolyomaviruses was well-supported with a bootstrap value of 97 per cent (LTAg, Supplementary File 2) and 100 per cent (VP1, Supplementary File 3). According to species demarcation criteria for polyomaviruses, a virus is considered as a separate species if the genetic distance from a member of the most closely related species is >15 per cent based on the LTAg coding sequence (Moens et al. 2017b). Since PPyV LTAg sequence was more than 35 per cent different (<65 per cent identity) to any of its closest relatives (Fig. 4), we propose that PPyV should be classified as a new species within the genus *Betapolyomavirus*.

Several different mammalian and avian polyomaviruses have been detected in New Zealand (Wang, Horner, and O’Keefe 2005; Wang et al. 2015; Rusinol et al. 2016), but this is the first report of a polyomavirus identified in a marsupial host. It is also the first description worldwide of a marsupial polyomavirus with a typical polyomavirus genome organisation and a mammalian-like LTAg. It has been previously suggested that marsupials may harbour polyomaviruses with avian-like T antigens based on the detection of bandicoot papillomatosis carcinomatosis viruses Types 1 and 2 in Australia, both of which appear to arise from recombination between an avian-like polyomavirus and a papillomavirus that was estimated to occur at least 10 million years ago (Woolford et al. 2007; Bennett et al. 2010). Recently discovered Tasmanian devil polyomaviruses also encode avian-like LTAg proteins (Chong et al. 2019). As these viruses were detected in faecal samples, it remains to be seen if they represent true marsupial viruses or contaminants that were present in the devils’ digestive tracts. Detection of rabbit haemorrhagic disease virus in the same study underscores the validity of this concern. Based on the results presented in this article, it appears that marsupials can also be infected with mammalian-like polyomaviruses. The development of a consistent taxonomy scheme for polyomaviruses has been hindered by the lack of understanding of polyomavirus evolution, partly due to the limited numbers of vertebrate hosts from which polyomavirus sequences have been identified (Buck et al. 2016). In this regard, our data provide a valuable contribution to our understanding of the evolutionary history of these viruses.

An intra-host divergence model for polyomavirus evolution has been recently proposed by Buck and others (Buck et al. 2016). According to this model, polyomavirus evolution is closely linked to the evolution of their hosts, but the viruses diverge at a slightly faster rate than their hosts from each other. As such, it would be expected that homologues of polyomaviruses that currently form small defined clades such as the WUKI group would be eventually found in other animal species (Buck et al. 2016), which is exactly what we have demonstrated in this study. If so, PPyV probably evolved from a polyomavirus that infected a common ancestor of rodents, humans, camelids, and marsupials before marsupials separated from other mammals about 160 million years ago (Hedges et al. 2015). Similar genetic distances between PPyV and its closest relatives within the WUKI clade (Fig. 4) suggest that these viruses evolved within their respective hosts at a similar rate. The diversity of hosts included in the WUKI clade, combined with similar genetic distances between PPyV and other viruses in this clade, makes the alternative theory that PPyV represents a recent cross-species transmission from one of those hosts less likely. Future discovery of further polyomaviruses from various animal species, including marsupials, will help to support or refute this theory.

Similar to all other polyomaviruses, the PPyV genome was predicted to contain three functional regions: early, late, and non-coding. The predicted early proteins (LTAg and STAg) contained all the conserved motifs described for other polyomaviruses (Moens et al. 2017a). The LXCXE motif binds the retinoblastoma (pRB) tumour suppressor protein and inhibits its function (Kim, Ahn, and Cho 2001; Singh et al. 2005). The Zn-ATPase domain of PPyV contains two highly conserved motifs (Table 3) that have been shown to be involved in the binding of the tumour suppressor protein p53 (An, Brodsky, and Pipas 2015). The LTAg is a complex protein with multiple functions in viral replication and host cell cycle progression (DeCaprio and Garcea 2013; Baez et al. 2017). PPyV lacked a CR1 motif (LXXLL) that is conserved in the N-terminus shared by the large and small TAg proteins of most polyomaviruses (Pipas 1992). This motif plays a role in many cellular protein–protein interactions that regulate transcription (Plevin, Mills, and Ikura 2005). There are a few polyomaviruses (e.g. goose haemorrhagic PyV or human polyomaviruses 6 and 7) whose large and small TAg proteins lack it, similar to PPyV and bandicoot papillomatosis carcinomatosis viruses 1 and 2. In most polyomaviruses, further copies of the LXXLL motif are also present in the C-terminal part of the LTAg, albeit their location and number vary between different viruses. The putative PPyV LTAg was predicted to contain two overlapping copies of the LXXLL motif at amino acids 574–579, similar to human polyomavirus 6. The role of all the above motifs in the virus interactions during infection of its marsupial host remains to be established.

Protein phosphatase 2A (PP2A) is essential in several cellular processes. It is a tumour suppressor protein that acts through dephosphorylation of a variety of signalling proteins (Sangodkar et al. 2016). Its interactions with STAg are believed to be important in polyomavirus-induced cell cycle progression and tumorigenesis (Mungre et al. 1994; Khalili, Sariyer, and Safak 2008). A zinc-binding domain with two conserved zinc-binding motifs (CXCXXC) was shown to play a role in inhibition of PP2A by STAg of SV40 by aiding the displacement of the control B subunit from the catalytic C and scaffolding A subunits (Mungre et al. 1994). A corresponding domain was present in the putative STAg of PPyV. In addition, a motif (LXXIXE) that has recently been described as required for binding to the B′ subunit of PP2A (Hertz et al. 2016) was identified at a site where CR1 was expected to be present. The PP2A-B′ was subsequently shown to also bind to several variants of this motif, with a consensus sequence [LM]xx[ILV]xE (Wu et al. 2017). Two more sequences consistent with this extended binding motif were identified in the putative LTAg of PPyV (Table 3), with the first also present in the N-terminus of the STAg. Hence, PPyV, similar to other polyomaviruses, is likely to interact with PP2A, but the exact nature of these interactions remains to be determined.

Two putative ORFs were identified upstream of ORFs predicted to encode viral structural proteins. The location of both ORFs and the charge of their putative protein products are consistent with a gene encoding a highly alkaline agnoprotein in the genomes of some polyomaviruses (Gerits and Moens 2012). BLAST searches did not identify any similarity between the two PPyV ORFs, or their predicted protein products, with any other nucleotide or protein sequences available in public databases. This is not too surprising, as the sizes and sequences of polyomavirus agnoproteins are highly variable (Gerits and Moens 2012). The ORF expressed from the traditional AUG codon was annotated as an agnoprotein gene in Fig. 1 and Table 2. It remains to be established whether or not either of these two ORFs is expressed during PPyV infection.

An ORF overlapping the beginning of the second exon of LTAg in a +1 frame was annotated as an ALTO-like gene based on the similar position of the ALTO gene in other polyomavirus genomes (Buck et al. 2016). Initially, the ALTO gene was identified only in a monophyletic group of viruses within the current *Alphapolyomavirus* genus referred to as the Almi clade, and it was proposed that this gene evolved *de novo* within that clade (Carter et al. 2013). Shorter ALTO-like sequences were later identified in polyomaviruses outside of the Almi group, which led to a suggestion that an ALTO-like gene may have been expressed by an ancestral polyomavirus and subsequently lost in some polyomavirus lineages (Buck et al. 2016). So far, a full-length ALTO gene has been shown to be expressed only in selected oncogenic alphaviruses, but the exact role of this protein remains unknown (van der Meijden and Feltkamp 2018).

In summary, we have identified a novel polyomavirus of possums. This is the first description of a typical (non-recombined) polyomavirus from a marsupial host with a non-avian-like LTAg, which adds to our knowledge of the diversity and evolution of this large group of viruses. It also represents an addition to a very limited number of marsupial viruses that have been identified so far.

## Supplementary Material

veac096_Supp

## Data Availability

The sequence of PPyV has been deposited in GenBank under the accession number ON614667. Raw NGS reads have been submitted to the NCBI SRA under the BioProject ID PRJNA861861 with BioSample numbers SAMN29929627, SAMN29929628, and SAMN29929629.
