# Supplementary material for: Identification of a novel polyomavirus from a marsupial host
Source: Virus Evol. 2022 Oct 6;8(2):veac096. doi: 10.1093/ve/veac096 (PMC9662318; doi:10.1093/ve/veac096)
Supplement: veac096_Supp [file veac096_supp.zip › suppl_data/Supplementary file 1_polyomavirus paper.docx]

| **Accession number** | **Nickname** | **Full virus name (ICTV)** | ***Species*** | ***Genus*** | **Full virus name (accession number)** |
| --- | --- | --- | --- | --- | --- |
| KR065722 | Wild boar | Sus scrofa polyomavirus 1 | *Alphapolyomavirus suis* | *Alphapolyomavirus* |  |
| MK443498 | Tree shrew | Tupaia belangeri polyomavirus | *Alphapolyomavirus tubelangeri* | *Alphapolyomavirus* | Tree shrew polyomavirus 1 |
| NC_001515 | Mouse BG | mouse polyomavirus strain BG | *Alphapolyomavirus muris* | *Alphapolyomavirus* |  |
| NC_001663 | Hamster | hamster polyomavirus 1 | *Alphapolyomavirus mauratus* | *Alphapolyomavirus* |  |
| NC_010277 | Human Mercel cell | Mercel cell polyomavirus | *Alphapolyomavirus quintihominis* | *Alphapolyomavirus* |  |
| NC_013439 | Orangutan Bo | Bornean orangutan polyomavirus | *Alphapolyomavirus ponpygmaeus* | *Alphapolyomavirus* |  |
| NC_014361 | Human TS | Trichodysplasia spinulosa-associated polyomavirus | *Alphapolyomavirus octihominis* | *Alphapolyomavirus* |  |
| NC_014743 | Chimpanzee Bob | chimpanzee polyomavirus | *Alphapolyomavirus panos* | *Alphapolyomavirus* | chimpanzee polyomavirus Bob |
| NC_015150 | Human 9 | human polyomavirus 9 | *Alphapolyomavirus nonihominis* | *Alphapolyomavirus* |  |
| NC_019844 | Vervet monkey 1 | vervet monkey polyomavirus 1 | *Alphapolyomavirus chlopygerythrus* | *Alphapolyomavirus* | Chlorocebus pygerythrus polyomavirus 1 |
| NC_019850 | Red colobus1 | Piliocolobus rufomitratus polyomavirus 1 | *Alphapolyomavirus pirufomitratus* | *Alphapolyomavirus* |  |
| NC_019851 | Crab-eating macaque | Macaca fascicularis polyomavirus 1 | *Alphapolyomavirus macacae* | *Alphapolyomavirus* |  |
| NC_019853 | Black spider monkey | Ateles paniscus polyomavirus 1 | *Alphapolyomavirus apaniscus* | *Alphapolyomavirus* |  |
| NC_019855 | Chimpanzee 4 | Pan troglodytes verus polyomavirus 3 | *Alphapolyomavirus quartipanos* | *Alphapolyomavirus* | Pan troglodytes verus polyomavirus 4 |
| NC_019856 | Chimpanzee 5 | Pan troglodytes verus polyomavirus 4 | *Alphapolyomavirus quintipanos* | *Alphapolyomavirus* | Pan troglodytes verus polyomavirus 5 |
| NC_019857 | Chimpanzee 6 | Pan troglodytes polyomavirus 5 | *Alphapolyomavirus sextipanos* | *Alphapolyomavirus* | Pan troglodytes polyomavirus 6 |
| NC_019858 | Chimpanzee 7 | Pan troglodytes schweinfurthii polyomavirus 2 | *Alphapolyomavirus septipanos* | *Alphapolyomavirus* | Pan troglodytes verus polyomavirus 7 |
| NC_020066 | Large-eared free-tailed bat 156 | Otomops polyomavirus 2 | *Alphapolyomavirus secomartiensseni* | *Alphapolyomavirus* | Otomops polyomavirus KY156 |
| NC_020067 | Heart-nosed bat | Cardioderma polyomavirus | *Alphapolyomavirus cardiodermae* | *Alphapolyomavirus* |  |
| NC_020068 | Straw-colored fruit bat | Eidolon helvum polyomavirus 1 | *Alphapolyomavirus eidoli* | *Alphapolyomavirus* |  |
| NC_020071 | Large-eared free-tailed bat 157 | Otomops polyomavirus 1 | *Alphapolyomavirus omartiensseni* | *Alphapolyomavirus* | Otomops polyomavirus KY156 |
| NC_023845 | Raccoon R45 | racoon polyomavirus | *Alphapolyomavirus procyonis* | *Alphapolyomavirus* | racoon polyomavirus strain R45 |
| NC_024118 | NJPyV | New Jersey polyomavirus | *Alphapolyomavirus terdecihominis* | *Alphapolyomavirus* |  |
| NC_025368 | Chimpanzee 1a | Pan troglodytes verus polyomavirus 1a | *Alphapolyomavirus secupanos* | *Alphapolyomavirus* |  |
| NC_025370 | Chimpanzee 3 | Pan troglodytes verus polyomavirus 2a | *Alphapolyomavirus tertipanos* | *Alphapolyomavirus* | Pan troglodytes verus polyomavirus 3 |
| NC_025380 | Gorilla | gorilla polyomavirus 1 | *Alphapolyomavirus gorillae* | *Alphapolyomavirus* |  |
| NC_025894 | Yellow baboon 1 | yellow baboon polyomavirus 1 | *Alphapolyomavirus pacynocephalus* | *Alphapolyomavirus* |  |
| NC_025898 | Vervet monkey 3 | vervet monkey polyomavirus 3 | *Alphapolyomavirus tertichlopygerythrus* | *Alphapolyomavirus* | Chlorocebus pygerythrus polyomavirus 3 |
| NC_026767 | Large flying fox | Bat polyomanvirus 5b-1 | *Alphapolyomavirus ptevampyrus* | *Alphapolyomavirus* |  |
| NC_026768 | Moluccan fruit bat 5a | Bat polyomavirus 5a | *Alphapolyomavirus dobsoniae* | *Alphapolyomavirus* |  |
| NC_027531 | Rat 1 | Rattus norvegicus polyomavirus 1 | *Alphapolyomavirus ranorvegicus* | *Alphapolyomavirus* |  |
| NC_028120 | Seba's short-tailed bat | bat polyomavirus 4b | *Alphapolyomavirus carolliae* | *Alphapolyomavirus* |  |
| NC_028123 | Velvety free-tailed bat | bat polyomavirus 3b | *Alphapolyomavirus molossi* | *Alphapolyomavirus* |  |
| NC_028127 | Orangutan Pi | Sumatran orangutan polyomavirus | *Alphapolyomavirus ponabelii* | *Alphapolyomavirus* |  |
| NC_034220 | Common bent-wing bat 1 | Miniopterus schreibersii polyomavirus 1 | *Alphapolyomavirus mischreibersii* | *Alphapolyomavirus* |  |
| NC_034221 | Common bent-wing bat 2 | Miniopterus schreibersii polyomavirus 2 | *Alphapolyomavirus secumischreibersii* | *Alphapolyomavirus* |  |
| NC_034253 | Human LI | LI polyomavirus | *Alphapolyomavirus quardecihominis* | *Alphapolyomavirus* |  |
| NC_038554 | Sulawesi flying fox 5b-2 | Bat polyomavirus 5b-2 | *Alphapolyomavirus acelebensis* | *Alphapolyomavirus* |  |
| NC_038555 | Flat-faced fruit bat A1055 | bat polyomavirus 3a-A1055 | *Alphapolyomavirus secarplanirostris* | *Alphapolyomavirus* | Artibeus planirostris polyomavirus 2 isolate A-1055 |
| NC_038557 | Little yellow-shouldered bat | bat polyomavirus 3a-B0454 | *Alphapolyomavirus sturnirae* | *Alphapolyomavirus* | Sturnira lilium polyomavirus 1 |
| NC_039051 | Western red colobus 2 | Piliocolobus badius polyomavirus 2 | *Alphapolyomavirus pibadius* | *Alphapolyomavirus* |  |
| KT987216 | Mouse Pt | mouse pneumotropic virus | *Betapolyomavirus secumuris* | *Betapolyomavirus* | Betapolyomavirus secumuris |
| KX574453 | Rat 2 | rat polyomavirus 2 | *Betapolyomavirus securanorvegicus* | *Betapolyomavirus* | Betapolyomavirus securanorvegicus strain PITT4 |
| MN994868 | Hare | Lepus polyomavirus 1 | *Betapolyomavirus leporis* | *Betapolyomavirus* |  |
| NC_001538 | Human BK | BK polyomavirus (Human polyomavirus 1) | *Betapolyomavirus hominis* | *Betapolyomavirus* |  |
| NC_001669 | SV40 | simian polyomavirus 40 | *Betapolyomavirus macacae* | *Betapolyomavirus* |  |
| NC_001699 | Human JC | JC polyomavirus | *Betapolyomavirus secuhominis* | *Betapolyomavirus* |  |
| NC_009238 | Human KI | KI polyomavirus | *Betapolyomavirus tertihominis* | *Betapolyomavirus* |  |
| NC_009539 | Human WU | WU polyomavirus | *Betapolyomavirus quartihominis* | *Betapolyomavirus* |  |
| NC_009951 | Black-capped squirrel monkey | squirrel monkey polyomavirus | *Betapolyomavirus saboliviensis* | *Betapolyomavirus* |  |
| NC_011310 | Mouse-eared bat | Myotis polyomavirus | *Betapolyomavirus myolucifugus* | *Betapolyomavirus* |  |
| NC_013796 | Sea lion | California sea lion polyomavirus 1 | *Betapolyomavirus zacalifornianus* | *Betapolyomavirus* |  |
| NC_017982 | Horse | equine polyomavirus | *Betapolyomavirus equi* | *Betapolyomavirus* | Equus caballus polyomavirus 1 |
| NC_019854 | White-fronted capuchin | Cebus albifrons polyomavirus 1 | *Betapolyomavirus calbifrons* | *Betapolyomavirus* |  |
| NC_020069 | African long-fingered bat | Miniopterus africanus polyomavirus 1 | *Betapolyomavirus mafricanus* | *Betapolyomavirus* |  |
| NC_020070 | Naked-backed bat | Pteronotus davyi polyomavirus 1 | *Betapolyomavirus ptedavyi* | *Betapolyomavirus* |  |
| NC_022519 | Elephant | African elephant polyomavirus 1 | *Betapolyomavirus elephanti* | *Betapolyomavirus* |  |
| NC_025259 | Sea otter | sea otter polyomavirus | *Betapolyomavirus enhydrae* | *Betapolyomavirus* |  |
| NC_025892 | Red-eared guenon | Cercopithecus erythrotis polyomavirus 1 | *Betapolyomavirus cercopitheci* | *Betapolyomavirus* |  |
| NC_025895 | Multimammate mouse1 | Mastomys polyomavirus | *Betapolyomavirus mastomysis* | *Betapolyomavirus* |  |
| NC_025896 | Vervet monkey 2 | vervet monkey polyomavirus 2 | *Betapolyomavirus secuchlopygerythrus* | *Betapolyomavirus* | Chlorocebus pygerythrus polyomavirus 2 |
| NC_025897 | Yellow baboon2 | yellow baboon polyomavirus 2 | *Betapolyomavirus secupacynocephalus* | *Betapolyomavirus* |  |
| NC_026473 | Badger | Meles meles polyomavirus 1 | *Betapolyomavirus meletis* | *Betapolyomavirus* |  |
| NC_026762 | Sulawesi flying fox 6a | Bat polyomavirus 6a | *Betapolyomavirus secacelebensis* | *Betapolyomavirus* |  |
| NC_026769 | Moluccan fruit bat 6c | Bat polyomavirus 6c | *Betapolyomavirus tertidobsoniae* | *Betapolyomavirus* |  |
| NC_026770 | Moluccan fruit bat 6b | Bat polyomavirus 6b | *Betapolyomavirus secudobsoniae* | *Betapolyomavirus* |  |
| NC_028117 | Bank vole | Myodes glareolus polyomavirus 1 | *Betapolyomavirus myoglareolus* | *Betapolyomavirus* |  |
| NC_028119 | Common vole | Microtus arvalis polyomavirus 1 | *Betapolyomavirus marvalis* | *Betapolyomavirus* |  |
| NC_028121 | Mustached bat | bat polyomavirus 2b | *Betapolyomavirus pteparnellii* | *Betapolyomavirus* |  |
| NC_028122 | Vampire bat | bat polyomavirus 2a | *Betapolyomavirus desrotundus* | *Betapolyomavirus* |  |
| NC_028635 | Chimpanzee 8 | Pan troglodytes verus polyomavirus 8 | *Betapolyomavirus octipanos* | *Betapolyomavirus* |  |
| NC_032120 | Wendell seal | Leptonychotes weddellii polyomavirus 1 | *Betapolyomavirus lepweddellii* | *Betapolyomavirus* |  |
| NC_034219 | Egyptian fruit bat 1 | Rousettus aegyptiacus polyomavirus 1 | *Betapolyomavirus raegyptiacus* | *Betapolyomavirus* |  |
| NC_034251 | Alpaca | alpaca polyomavirus | *Betapolyomavirus vicugnae* | *Betapolyomavirus* | Vicugna pacos polyomavirus 1 |
| NC_034456 | Dog | Canis familiaris polyomavirus 1 | *Betapolyomavirus canis* | *Betapolyomavirus* |  |
| NC_038559 | Common squirrel monkey | Saimiri sciureus polyomavirus 1 | *Betapolyomavirus sasciureus* | *Betapolyomavirus* |  |
| KY549442 | Raccoon 2 | Raccoon-associated polyomavirus 2 | *Deltapolyomavirus secuprocyonis* | *Deltapolyomavirus* |  |
| KY612371 | Giant panda | giant panda polyomavirus | *Deltapolyomavirus ailuropodae* | *Deltapolyomavirus* |  |
| MG701355 | Wolf | Canis lupus polyomavirus 1 | *Deltapolyomavirus canis* | *Deltapolyomavirus* |  |
| NC_014406 | Human 6 | human polyomavirus 6 | *Deltapolyomavirus sextihominis* | *Deltapolyomavirus* |  |
| NC_014407 | Human 7 | human polyomavirus 7 | *Deltapolyomavirus septihominis* | *Deltapolyomavirus* |  |
| NC_018102 | Human MW | MW polyomavirus | *Deltapolyomavirus decihominis* | *Deltapolyomavirus* |  |
| NC_020106 | Human STL | STL polyomavirus | *Deltapolyomavirus undecihominis* | *Deltapolyomavirus* |  |
| MG654479 | Wild goat | Capra aegagrus polyomavirus 1 | *Epsilonpolyomavirus caprae* | *Epsilonpolyomavirus* |  |
| NC_001442 | Bovine | bovine polyomavirus | *Epsilonpolyomavirus bovis* | *Epsilonpolyomavirus* |  |
| NC_040634 | Red river hog | Potamochoerus porcus polyomavirus 1 | *Epsilonpolyomavirus poporcus* | *Epsilonpolyomavirus* |  |
| NC_026244 | Giant guitarfish |  | *Etapolyomavirus rhyndjiddensis* | *Etapolyomavirus* | Rhynchobatus djiddensis polyomavirus 1 |
| NC_004764 | Budgerigar | budgerigar fledgling disease virus - 1 | *Gammapolyomavirus avis* | *Gammapolyomavirus* |  |
| NC_004800 | Goose | goose hemorrhagic polyomavirus | *Gammapolyomavirus anseris* | *Gammapolyomavirus* |  |
| NC_007922 | Crow | crow polyomavirus | *Gammapolyomavirus corvi* | *Gammapolyomavirus* |  |
| NC_007923 | Bullfinch | finch polyomavirus | *Gammapolyomavirus pypyrrhula* | *Gammapolyomavirus* |  |
| NC_017085 | Canary | canary polyomavirus | *Gammapolyomavirus secanaria* | *Gammapolyomavirus* |  |
| NC_023008 | Butcherbird | butcherbird polyomavirus | *Gammapolyomavirus cratorquatus* | *Gammapolyomavirus* |  |
| NC_026141 | Adelie penguin | Adelie penguin polyomavirus | *Gammapolyomavirus padeliae* | *Gammapolyomavirus* |  |
| NC_039052 | Gouldian finch | Erythrura gouldiae polyomavirus 1 | *Gammapolyomavirus egouldiae* | *Gammapolyomavirus* |  |
| NC_039053 | White-headed munia finch | Hungarian finch polyomavirus | *Gammapolyomavirus lonmaja* | *Gammapolyomavirus* | Lonchura maja polyomavirus 1 |
| MG800627 | Emerald rockcod |  | *Thetapolyomavirus trebernacchii* | *Thetapolyomavirus* | Trematomus bernacchii polyomavirus 1 |
| NC_025790 | Black sea bass |  | *Thetapolyomavirus censtriata* | *Thetapolyomavirus* | black sea bass polyomavirus 1 |
| NC_026944 | Ray-finned fish |  | *Thetapolyomavirus trepennellii* | *Thetapolyomavirus* | Trematomus pennellii polyomavirus 1 |
| NC_030838 | Sea bream |  | *Thetapolyomavirus spari* | *Thetapolyomavirus* | Sparus aurata polyomavirus 1 |
| MK513523 | Tasmanian Devil |  | *unclassified* | *unclassified* | Tasmanian devil-associated polyomavirus 1 |
| MT150088 | Rabbit |  | *unclassified* | *unclassified* | rabbit polyomavirus |
| MT457856 | Magpie |  | *unclassified* | *unclassified* | magpie polyomavirus |
| MW054655 | Cat |  | *unclassified* | *unclassified* | cat-associated polyomavirus |
| NC_010107 | Bandicoot 1 |  | *unclassified* | *unclassified* | Bandicoot papillomatosis carcinomatosis virus type 1 |
| NC_010817 | Bandicoot 2 |  | *unclassified* | *unclassified* | Bandicoot papillomatosis carcinomatosis virus type 2 |
| NC_026942 | Sheep |  | *unclassified* | *unclassified* | Sheep polyomavirus 1 |
| NC_055162 | Common shrew |  | *unclassified* | *unclassified* | Sorex araneus polyomavirus 1 |
| NC_055163 | Crowned shrew |  | *unclassified* | *unclassified* | Sorex coronatus polyomavirus 1 |
| NC_055164 | Pygmy shrew |  | *unclassified* | *unclassified* | Sorex minutus polyomavirus 1 |
| NC_055488 | Mouse 3 |  | *unclassified* | *unclassified* | Mus musculus polyomavirus 3 |
| ON614667 | Possum |  | *unclassified* | *unclassified* | possum polyomavirus |
| NC_025899 | Dolphin | dolphin polyomavirus 1 | *Zetapolyomavirus delphini* | *zetapolyomavirus* | Delphinus delphis polyomavirus 1 |
