# Supplementary figures and images for: Identification of a novel polyomavirus from a marsupial host
Source: Virus Evol. 2022 Oct 6;8(2):veac096. doi: 10.1093/ve/veac096 (PMC9662318; doi:10.1093/ve/veac096)

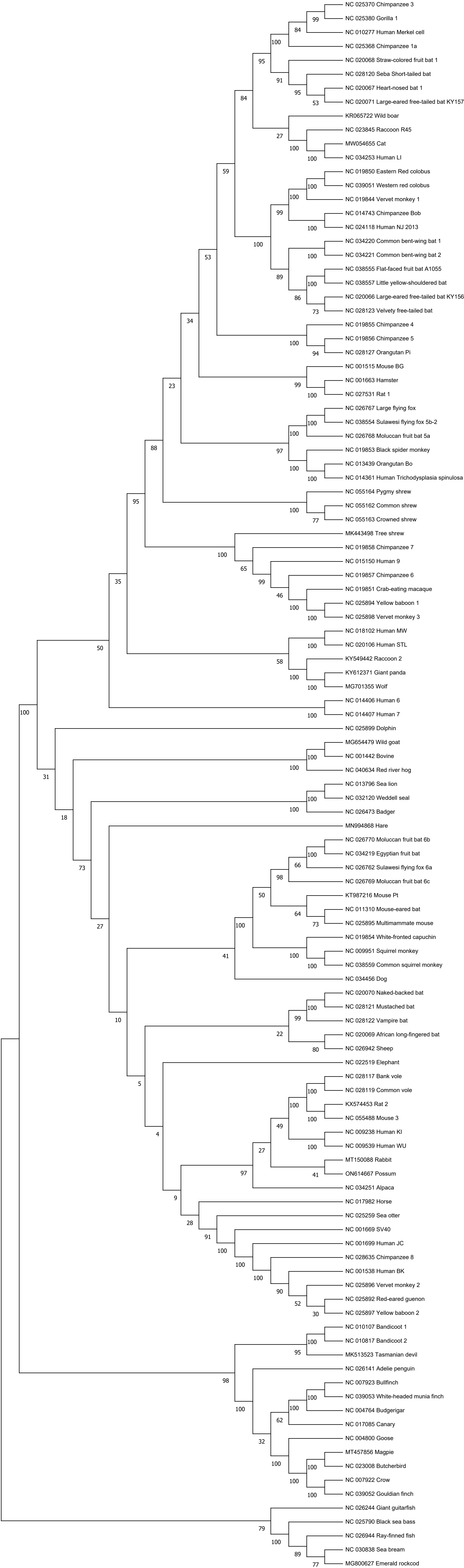

Supplement: veac096_Supp [file veac096_supp.zip › suppl_data/Supplementary file 2 LTAg113aa_with bootstrap_29Aug.pdf]

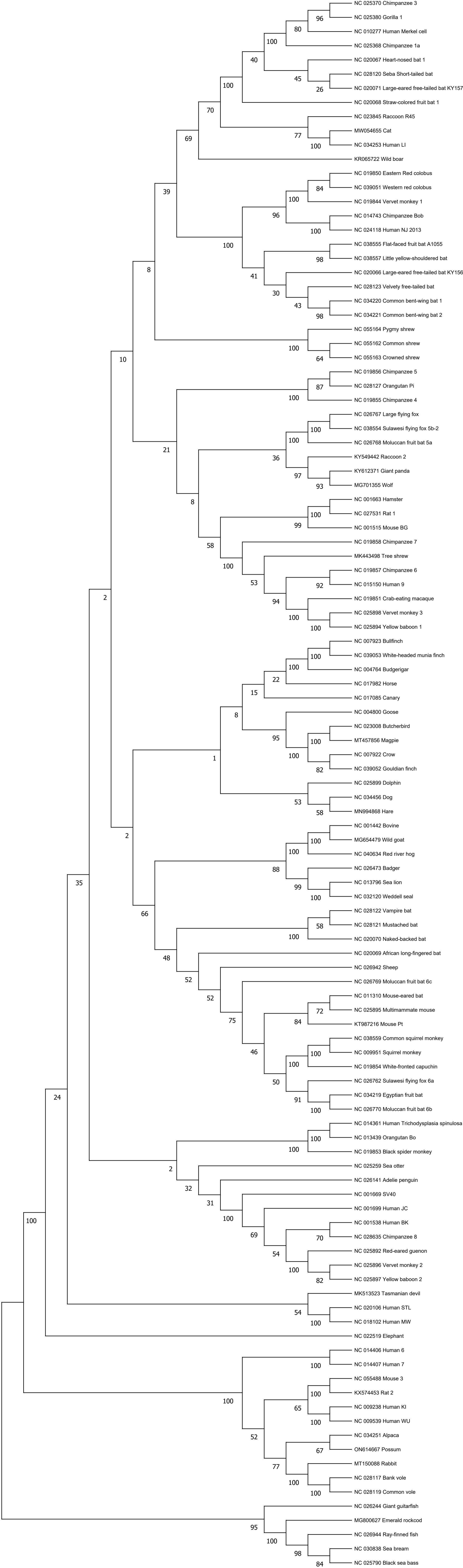

Supplement: veac096_Supp [file veac096_supp.zip › suppl_data/Supplementary File 3 VP1 with bootstrap values.pdf]
